# Change in Employment Status Due to the COVID-19 Pandemic, SNAP Participation, and Household Food Insecurity among Black and Latino Adults in Illinois

**DOI:** 10.3390/nu14081581

**Published:** 2022-04-11

**Authors:** Chelsea R. Singleton, Olufemi Fabusoro, Margarita Teran-Garcia, Sandraluz Lara-Cinisomo

**Affiliations:** 1 Department of Social, Behavioral, and Population Sciences, School of Public Health and Tropical Medicine, Tulane University, New Orleans, LA 70112, USA; 2 Division of Nutritional Sciences, University of Illinois at Urbana-Champaign, Urbana, IL 61801, USA; okf2@illinois.edu (O.F.); teranmd@illinois.edu (M.T.-G.); laracini@illinois.edu (S.L.-C.); 3 Illinois Extension, University of Illinois at Urbana-Champaign, Urbana, IL 61801, USA; 4 Department of Kinesiology and Community Health, University of Illinois at Urbana-Champaign, Champaign, IL 61820, USA

**Keywords:** food security, unemployment, COVID-19, SNAP, disparities, Illinois

## Abstract

The onset of the COVID-19 pandemic resulted in record-high unemployment rates. Black and Latino adults experienced disproportionately higher rates of unemployment. We aimed to examine associations between pandemic-related employment status change and household food insecurity among an economically diverse sample of Black and Latino adults in Illinois during the early months of the COVID-19 pandemic. Furthermore, we evaluated the significance of Supplemental Nutrition Assistance Program (SNAP) participation to determine if it modified associations. We analyzed cross-sectional data collected from 1,809 Black and Latino adults in two waves: May 2020 and June/July 2020. Participants listed their change in employment status as “lost job entirely”, “employed, but paid hours reduced”, “employed, but anticipate job lost”, or “no change”. Participants self-reported their SNAP status and completed the USDA’s six item U.S. Food Security Module to report household food security status. We used logistic regression to assess the significance of associations after controlling for socio-demographics. Approximately 15.5% of participants lost their job entirely, 25.2% were SNAP participants, and 51.8% reported low food security (LFS). All changes in employment were significantly associated with increased odds of LFS after adjusting for socio-demographics. SNAP participants who lost their job had higher odds of LFS (OR: 4.69; 95% CI: 2.69–8.17) compared to non-participants who lost their job (OR: 2.97; 95%: 1.95–4.52). In summary, we observed strong associations between changes in employment and household food insecurity, particularly among SNAP participants, which underscores the pandemic’s impact on low-income and minority populations.

## 1. Introduction

The ongoing COVID-19 pandemic has proven to be an unprecedented public health crisis capable of affecting all facets of everyday life [1]. The initial wave of widespread infection in the U.S. occurred in the early months of 2020 and resulted in a nearly instantaneous pause in social interaction and economic productivity [2,3]. As businesses and organizations closed or altered their operations to comply with state and local policies, many American workers faced the challenge of unexpected job loss [4]. Data from the Congressional Research Service revealed that the national unemployment rate reached 14.8% in April 2020, which was the highest rate ever recorded since data tracking on employment began in 1948 [5].

Employment is an important part of life as it provides financial stability and access to vital resources such as housing, healthcare, and food. Several recent studies have reported disparities in unemployment by socioeconomic status and race/ethnicity early in the pandemic in the U.S. [5,6,7]. Low-income individuals were more likely to lose their job compared to higher income individuals [5]. Black and Latino adults had significantly higher unemployment rates in April 2020 compared to White adults [5]. Furthermore, the Black-White gap in unemployment increased by the end of 2020 because the rate among White adults decreased late in the year while the rate among Black adults remained stagnant [7]. 

These statistics on disparities are particularly concerning given the well-established association between unemployment and food insecurity. Like unemployment rates, food insecurity rates also increased due to economic fallout from the pandemic [8,9,10,11]. Feeding America estimated that 1 in 6 adults and 1 in 4 children in the U.S. experienced food insecurity in 2020 [8]. As expected, several recent studies have linked unemployment to food security status [12,13,14,15]. These studies have mostly reported that pandemic-related job loss is associated with higher levels of food insecurity [12,13,14,15,16,17]. Despite the consistency of these findings, there is little understanding of how participating in federal nutrition assistance programs, specifically the Supplemental Nutrition Assistance Program (SNAP), influenced the relationship between job loss and food insecurity in the early months of the pandemic.

SNAP participation provides positive benefits to low-income adults and children, which includes improvements in nutritional outcomes, better academic performance, and lower healthcare costs [18,19]. Nevertheless, studies have shown that SNAP participants are significantly different from non-participants regarding socio-demographics and nutritional vulnerability. SNAP participants are more likely to be young, female, a racial/ethnic minority, a single parent/caregiver, and an hourly/minimum wage worker [20,21]. In addition, SNAP participants, on average, have poorer diet quality and higher rates of food insecurity compared to both income-eligible and ineligible non-participants [22]. Given the existing financial and nutritional vulnerability of SNAP participants, it is possible that pandemic-related job loss affected their food security status differently than non-participants. 

The research study aimed to evaluate associations between pandemic-related change in employment, SNAP participation, and household food security status among a large and economically diverse sample of Black and Latino adults residing in Illinois during the early months of the COVID-19 pandemic. A main goal of this study was to determine if the odds of reporting low food security status given an individual’s pandemic-related change in employment status varied by SNAP participation status. The socioeconomic consequences of the pandemic unveiled, and worsened, several inequities in health determinants that historically affected socially disadvantaged populations such as low-income individuals and racial/ethnic minorities [23]. Given this knowledge, there is a need to determine if the pandemic worsened gaps in household food insecurity by SNAP participation status. We hypothesized that complete job loss would be associated with higher odds of low food security among study participants. Furthermore, given the well-documented differences in pre-pandemic food insecurity rates, associations between pandemic-related job loss and low food security status would vary significantly between SNAP participants and non-participants.

## 2. Materials and Methods

### 2.1. Data Source and Participants

To study associations between change in employment status, SNAP participation, and household food security, we conducted a secondary data analysis of cross-sectional data collected from a large and diverse sample of Illinois residents. The Center for Social and Behavioral Sciences (CSBS) at the University of Illinois at Urbana-Champaign (UIUC) worked with two private research firms to design and implement the survey online using the REDCap web application. They collected the data at two time points (i.e., waves): April 2020 and June/July 2020. The overarching aim of the survey was to measure the early effects of the COVID-19 pandemic on Illinois residents. Eligible members of each company’s survey panel received invitations to complete the survey. Both firms offered the survey in English and Spanish to ensure inclusivity. 

All members of each company’s survey panel aged 18 or older and that were Illinois residents were eligible to participate. A volunteer sample of 4437 adults participated in the only survey: wave one (N = 2294) and wave two (N = 2143). For the current study, we only included individuals who self-identified as Latino or Black (non-Latino). Approximately 1809 participants (~41% of the total sample) met this inclusion criterion and were included in the analytical sample. We excluded all other survey participants from the analysis. The Institutional Review Board (IRB) at UIUC approved the study. All participants provided informed consent prior to starting the online survey.

### 2.2. Variables

Household food security status served as the outcome variable. We measured household food security status using the U.S. Department of Agriculture’s (USDA) six-item food security module [24]. The module is a valid and reliable tool that features six questions designed to assess the extent of a household’s food insecurity in the past 12 months because of financial constraints [25]. Depending on an individual’s responses, scores are assigned on a scale of zero (low) to 6 (high), which reflect food security/marginal security (score = 0–1), low food security (score = 2–4), and very low food security (score = 5 or 6). 

The primary explanatory variables were pandemic-related change in employment status and SNAP participation status. To assess employment status change, participants responded to the question “Have you been put on leave or otherwise had your paid hours reduced because of the Coronavirus pandemic?” They selected one of the following four response options: “Yes, I lost my job entirely”, “Yes, I had my paid hours reduced”, “No, but I expect to lose employment or paid hours in the next few weeks”, and “No, my employment hasn’t changed”. Participants self-reported their SNAP participation status by answering yes or no if they received SNAP benefits/food stamps. 

Covariates of interest included age (years), race/ethnicity (non-Latino Black vs. Latino), sex (female vs. other), educational level (≤high school diploma/GED, some college/vocational degree, college degree, or graduate degree), pre-pandemic annual income (<$20,000, $20,000–$49,999, $50,000–$99,999, or ≥$100,000), spouse/partner status (yes or no), and children status (yes or no). The other group for sex included participants who self-identified as male or non-binary (*n* = 15). For spouse/partner status, the participant’s spouse or partner had to be residing in their home. For children status, the participant had to have at least one child under age 18 residing in their home.

### 2.3. Statistical Analysis

We used SAS version 9.4 to perform the statistical analysis [26]. We calculated means and frequencies for variables of interest among all survey participants and stratified by three groups representing household food security status: food secure/marginal security, low food security, and very low food security. To determine if descriptive statistics varied by food security group, we ran chi-square test of independence (categorical variables) and one-way analysis of variance (ANOVA) (continuous variables). *p* values < 0.05 were considered statistically significant. We examined unadjusted and adjusted logistics regression models to evaluate associations between pandemic-related employment status change and odds of reporting low food security status. For these regression models, “low food security status” and “very low food security status” served as the outcome variables. “Low food security status” reflected individuals experiencing low or very low food security status (*n* = 937). The food secure/marginal security group served as the reference group in all logistic regression models. Since participants completed the surveys in two waves, we accounted for the wave number in all models. Adjusted models also include socio-demographic variables including age, sex, race/ethnicity, education level, pre-pandemic annual income, spouse status, and children status. 

We utilized two different techniques to determine if associations between change in employment status and household food security status varied by SNAP participation status: interaction terms and stratified logistic regression models. Terms modeling the interaction between pandemic-related employment status change and SNAP participation were incorporated in an adjusted logistic regression model. For these models, we modeled individuals who were not SNAP participants and did not report any change in employment as the reference group. Thus, we compared all other combinations of employment status change x SNAP participation status to this reference group. Stratified logistic regression models evaluated associations between employment status change and the two outcome variables among SNAP participants and non-participants, separately. For all logistic regression models, we considered all 95% confidence intervals that did not include the null value of 1.0 to be statistically significant.

## 3. Results

Table 1 displays descriptive characteristics of the survey participants stratified by household food security status. Approximately 48.2%, 35.2%, and 16.6% of the 1809 participants reported experiencing food security/marginal security, low food security (LFS), and very low food security (VLFS), respectively. The mean age was 37.1 (±15.4). The majority of participants were female (63.9%) and non-Latino Black (60.1%). Only 36.6% of participants lived with a spouse/partner, and only 35.8% lived with at least one child. The distribution of education level and pre-pandemic annual income was diverse. About 40% of the participants had at least a college degree, with 13% having a graduate or professional degree. Fourteen percent of participants self-reported their pre-pandemic annual income at ≥$100,000. About 25% of participants self-reported that they, or a member of their household, were receiving SNAP benefits. Furthermore, 36.1% reported a pandemic-related change in employment status; 15.5% lost their job, and 20.6% had their paid hours reduced. Another 14% reported that they anticipated job loss in the upcoming weeks to months. Chi-square and ANOVA tests revealed that all explanatory variables of interest were associated with household food security status except race/ethnicity and spouse/partner status. Compared to food secure participants, there was a higher prevalence of younger adults, individuals living with children, individuals with less than a high school education, individuals making <$20,000/year, and individuals who lost their job entirely because of the pandemic among the participants experiencing VLFS. 

Table 2 and Table 3 report results from logistic regression models examining associations between pandemic-related change in employment status and the two outcome variables: LFS (Table 2) and VLFS (Table 3). Unadjusted logistic regression models presented in both tables revealed that change in employment status and SNAP participation status were significantly associated with higher odds of reporting LFS and VLFS. After adjusting for covariates, both variables retained their statistical significance. All three categories of employment status change were significantly associated with LFS and VLFS with complete job loss reporting the strongest associations. Compared to participants reporting no change in employment status, the odds of reporting LFS were about three times higher among participants who lost their job entirely (OR: 2.70; 95% CI: 1.91–3.81). The odds of reporting VLFS were about four times higher (OR: 3.61; 95% CI: 2.43–5.36) among participants who lost their job entirely. Individuals who reported that they had their paid hours reduced or anticipated job loss in the near future also had greater odds of reporting LFS and VLFS compared to those who had no change in employment status. 

The interaction terms presented in Table 2 and Table 3 describe how the interaction of the variables employment status change and SNAP participation influence odds of reporting LFS and VLFS. Individuals who reported no change in employment status AND were not SNAP participants served as the reference group. Among all of the employment status change × SNAP participation combinations, the group that experienced the highest odds of LFS and VLFS was individuals who lost their job entirely and were SNAP participants. Compared to the reference group, the odds of reporting LFS were nearly five times higher among those who lost their job entirely AND were SNAP participants (OR: 4.69; 95% CI: 2.69–8.17). The odds of reporting LFS were also higher among individuals who lost their job entirely but were not participating in SNAP (OR: 2.97; 95% CI: 1.95–4.52), but the association was not as strong. The models where VLFS served as the outcome variable revealed a similar pattern. Compared to the reference group, the odds of reporting VLFS were six times higher among participants who lost their job entirely and were SNAP participants (OR: 6.07; 95%: 3.38–10.90). Again, the odds of reporting VLFS were also higher among those who lost their job but were not SNAP participants (OR: 4.58; 95% CI: 2.74–7.67), but not as high as SNAP participants.

Table 4 includes results from stratified logistic regression models that examined associations between pandemic-related change in employment status and food security among SNAP participants and non-participants, separately. All models presented in this table were adjusted for covariates. Complete job loss was associated with higher odds of LFS and VLFS among SNAP participants and non-participants. The strength of this association was higher among non-participants compared to SNAP participants. For example, complete job loss was associated with nearly five times higher odds of VLFS among non-participants (OR: 4.46; 95% CI: 2.64–7.52) but only three times higher odds among SNAP participants (OR: 2.82; 95% CI: 1.46–5.45). Other categories of employment status change were either marginally significant or not significant among SNAP participants. All other categories of employment status change were significantly associated with higher odds of LFS and VLFS among non-participants.

## 4. Discussion

The objective of this study was to examine associations between pandemic-related changes in employment status, SNAP participation status, and household food security status among a large and economically diverse sample of Black and Latino adults residing in Illinois during the early months of the COVID-19 pandemic. Given the inequities in food insecurity observed by race/ethnicity since the onset of the pandemic [5,6,9,10,11], there is a need to improve the field’s understanding of (1) the effects of employment status change on food security on racial/ethnic minorities and (2) the differential impact of the pandemic given an individual’s SNAP participation status.

The results confirmed both our hypotheses. Complete job loss nearly tripled the odds of LFS and VLFS in our analytical sample. Participants who experienced reductions in paid work hours or anticipated job loss in the near future also had higher odds of LFS and VLFS compared to those who did not experience job loss. Thus, every category of the variable pandemic-related change in employment status, including anticipation of job loss, was associated with higher odds of household food insecurity. Nearly all other studies evaluating the relationship between pandemic-related unemployment and food insecurity reported significant findings [12,13,14,15]. Janda et al. (2021) found that job loss was correlated with becoming newly food insecure among a sample of low-income households living in Central Texas in 2020 [12]. Niles et al. (2020) reported tripled odds of household food insecurity among a statewide representative sample of adults living in Vermont in 2020 [15]. Lauren et al. (2021) did not find a connection between job loss and food insecurity in their national sample of adults surveyed online [16]. However, it is important to note that their survey was conducted in March 2020, at a time when many businesses and schools were still transitioning away from normal operations. The consistency in results across geographic areas and populations strengthens the body of evidence that the economic shutdown was detrimental to U.S. households. As the pandemic enters its second year, more research is needed to describe the longitudinal relationship between changes in employment and household food security in the U.S., and, ultimately, the impact of food insecurity on diet and health among historically disadvantaged populations. 

Our findings suggest that associations varied by SNAP participation status. The strength of association between complete job loss and both LFS and VLFS was stronger among SNAP participants than non-participants. This could be due to the elevated risk of food insecurity that SNAP participants in our sample were already experiencing prior to the economic shutdown. Unfortunately, we did not collect data on participants’ pre-pandemic food security status to ascertain its effects. We also did not collect data longitudinally throughout the remaining months of 2020 to determine if differences in risk between SNAP participants and non-participants declined over time. Nevertheless, these findings highlight the long-standing evidence that negative changes to household income or job status increases the risk of food insecurity among SNAP participants [27]. 

To our knowledge, only one study has examined the intersection of pandemic-related unemployment, SNAP participation, and food insecurity. Molitor et al. (2021) evaluated change in food insecurity status before and after the onset of the pandemic among a large sample of low-income Latina mothers living in California [17]. They found that the prevalence of VLFS among the women declined from 19.3% to 15.3% between their two measuring periods: before (November 2019–March 2020) and after (August 2020–September 2020) [17]. Furthermore, they reported that SNAP participation was associated with a lower prevalence of VLFS [17]. Although not related to the SNAP program, Raifman et al. (2021) reported that low-income adults in America who received unemployment insurance benefits experienced a significant decline in food insecurity risk from April 2020 to November 2020 [13]. The findings from these two studies demonstrate the health and nutritional benefits of public assistance programs during a time of public health crisis.

It is important to note that individuals who lost their job but were not receiving SNAP benefits also had an elevated risk of LFS and VLFS. When stratified by SNAP participation status, we saw a dose response relationship between change in employment status and VLFS among non-participants. The stark rise in unemployment and food insecurity proved to be an unprecedented challenge in 2020. In response to the rise in need and demand for food assistance, the USDA introduced new legislation (e.g., FFCRA Act, CARES Act) that allowed states to adjust program operations, increase SNAP benefits, and create temporary programs that provided meals to nutritionally-vulnerable children while schools remained closed [28,29]. This legislation was integral to many individuals and families in the U.S., particularly those who historically have been socially and economically disadvantaged like racial/ethnic minorities. The study by Niles et al. (2020) reported that food assistance program participation, particularly SNAP participation, increased significantly among newly food insecure adults [15]. As the pandemic moves into its second year, demand for food assistance programs is likely to rise [30]. To ensure all individuals have the opportunity to benefit from these programs, state and local efforts are needed to inform existing, new, and prospective participants, especially those who are socially and economically disadvantaged, about important provisional changes to the program (i.e., changes in eligibility status, changes in application process, increases in benefit allotment, etc.). 

### Strengths and Limitations

This study has several strengths. First, the sample included racially, ethnically and linguistically diverse adults. The sample also represented a range of ages, education levels, and pre-pandemic income levels. Second, the study used validated measures to assess food security status, specifically the U.S. Food Security Module. Third, the data were collected shortly after the beginning of the pandemic, allowing for an early assessment of the pandemic’s effects on food security in Illinois. Last, the analysis compared participants and non-participants of the SNAP program, which enabled us to take a more nuanced look at the effects of pandemic-related employment changes on food security.

A key limitation of this study is the reliance on self-reported data rather than objective data, which would have allowed us to verify the information reported on the survey. SNAP participation is best determined using objective data to categorize participants’ eligibility [31]. We could not verify whether participants were receiving SNAP benefits at the time of the survey, which would have enabled us to address any reporting errors. In addition, pre-pandemic income was not verifiable, making it impossible to confirm that self-reported pre-pandemic incomes were accurate. Thus, we could only ascertain SNAP participation status based on self-report. Because of the wide-range of income levels among individuals not receiving SNAP benefits, researchers often compare SNAP participants to income-eligible and income-ineligible non-participants separately [21,22]. This allows researchers to address the issue of confounding by income when comparing SNAP participants to non-participants. Unfortunately, the survey did not allow us to separate non-participants by eligibility status and we could not properly ascertain eligibility status.

Another limitation is the cross-sectional design, which prevented us from establishing temporality and directionality between the independent and dependent variables. We were not able to identify each participant’s employment type or whether they had steady employment at the start of the pandemic. This information would have allowed us to clarify who had a higher risk of job loss. We did not have information on food security status for the participants prior to the pandemic. Therefore, we could not identify which participants were newly food insecure because of the pandemic. Finally, the sample included only non-Latino Black and Latino adults living in Illinois, which limits the generalizability of the findings. Future studies should enroll national samples of Black and Latino individuals and members of other racial and ethnic groups.

## 5. Conclusions

In summary, the results from this study revealed the early and significant consequences of the COVID-19 pandemic on racial/ethnic minorities in Illinois. Half of the Black and Latino adults participating in our study reported food insecurity. The COVID-19 pandemic has unveiled, and worsened, several inequities in health and health determinants that disproportionately affect racial/ethnic minorities in the U.S. [6,7]. In addition to experiencing higher rates of unemployment and food insecurity, Black and Latino adults have experienced higher rates of infection and death from coronavirus compared to White adults [32,33]. To achieve nutrition equity in the U.S., researchers, practitioners, and policy makers must continue developing and evaluating programs to combat the health and nutritional consequences of the pandemic among historically disadvantaged populations, which includes racial/ethnic minorities.

Our study also revealed that participants who experienced pandemic-related job loss had high odds of reporting food insecurity, with those participating in SNAP being highly vulnerable. This confirms our primary hypothesis that job loss would be associated with increased risk of food insecurity. Study participants who were not receiving SNAP benefits and experienced job loss also had an elevated risk of food insecurity. Thus, there is a need to provide support to individuals and families that are consistently and newly food insecure. The expanded USDA programming mentioned above is a good start and should continue as the pandemic wears on. Food assistance can help mitigate the short-term and long-term physical, mental, and emotional consequences of food insecurity during a pandemic. [15,34]. Thus, future research and programs should (1) continue to document the interplay of employment, federal food assistance, and food security, and (2) strengthen and allocate resources to support those most at risk for food insecurity in the upcoming months of the COVID-19 pandemic.

## Figures and Tables

**Table 1:** Demographic Characteristics of Survey Participants by Food Security Status.

Variable	All Participants *n* = 1809	Food Secure/ Marginal Security 872 (48.2)	Low Food Security 637 (35.2)	Very Low Food Security 300 (16.6)	*p* Value ^a^
Age (years), mean (±sd)	37.1 (±15.4)	40.9 (±16.2)	31.9 (±13.0)	35.1 (±13.8)	<0.0001
Sex, *n* (%)					0.0005
Female	1150 (63.9)	581 (66.8)	365 (57.8)	204 (68.0)	
Other ^b^	651 (36.2)	289 (33.2)	266 (42.2)	96 (32.0)	
Race/Ethnicity, *n* (%)					0.65
Latino	722 (39.9)	346 (39.7)	262 (41.1)	114 (38.0)	
Non-Latino Black	1087 (60.1)	529 (60.3)	375 (58.9)	189 (62.0)	
Lives with Spouse/Partner, *n* (%)					0.10
Yes	662 (36.6)	340 (39.0)	204 (33.6)	102 (35.1)	
No	1147 (63.4)	533 (61.0)	404 (66.5)	189 (64.9)	
Lives with Children, *n* (%)					0.02
Yes	648 (35.8)	306 (35.1)	214 (33.6)	128 (42.7)	
No	1161 (64.2)	566 (64.9)	423 (66.4)	172 (57.3)	
Education Level, *n* (%)					<0.0001
≤HS Diploma/GED	510 (28.4)	210 (24.2)	198 (31.3)	102 (34.2)	
Some College/Vocational	595 (33.1)	275 (31.7)	221 (34.9)	99 (33.2)	
College Degree	459 (25.5)	243 (28.0)	146 (23.1)	70 (23.5)	
Graduate/Professional Degree	234 (13.0)	139 (16.0)	68 (10.7)	27 (9.1)	
Pre-Pandemic Annual Income, *n* (%)					
<$20,000	455 (26.6)	156 (19.1)	195 (32.5)	104 (35.7)	<0.0001
$20,000–$49,999	543 (31.8)	267 (32.6)	196 (32.6)	80 (27.5)	
$50,000–$99,999	477 (27.9)	269 (32.9)	139 (23.1)	69 (23.7)	
≥$100,000	235 (13.7)	126 (15.4)	71 (11.8)	38 (13.1)	
Current SNAP Participant, *n* (%)					<0.0001
Yes	456 (25.2)	167 (19.1)	180 (28.3)	109 (36.3)	
No	1353 (74.8)	705 (80.9)	457 (71.7)	191 (63.7)	
Employment Status Change Due to COVID-19 Pandemic, *n* (%)					<0.0001
Lost Job Entirely	281 (15.5)	90 (10.3)	99 (15.5)	92 (30.7)	
Paid Hours Reduced	372 (20.6)	136 (15.6)	156 (24.5)	80 (26.7)	
Anticipate Job Lost	253 (14.0)	74 (8.5)	145 (22.8)	34 (11.3)	
No Change	903 (49.9)	572 (65.6)	237 (37.2)	94 (31.3)	

HS: High School; SNAP: Supplemental Nutrition Assistance Program; sd: standard deviation. Note: Frequencies in cells may not sum to total sample size due to missing data. ^a^ *p*-values calculated with a chi-square test of independence (categorical variables) or one-way ANOVA (continuous variables). *p*-values < 0.05 are considered statistically significant. ^b^ Other group for sex include participants who self-identified as male or non-binary.

**Table 2:** Associations between Employment Status Change during COVID-19 Pandemic, SNAP Participation, and Low Food Security ^a^.

Variable	Unadjusted Models ^b^ OR (95% CI)	Adjusted Model 1 ^c^ OR (95% CI)	Adjusted Model 2 ^d^ OR (95% CI)
Employment Status Change Due to COVID-19 Pandemic:			
Lost Job Entirely	3.67 (2.76–4.88)	2.70 (1.91–3.81)	2.97 (1.95–4.52)
Paid Hours Reduced	3.00 (2.33–3.85)	2.07 (1.53–2.81)	2.62 (1.85–3.70)
Anticipate Job Loss	4.18 (3.09–5.66)	2.54 (1.73–3.73)	2.71 (1.77–4.15)
No Change	REF	REF	REF
Current SNAP Participant:			
Yes	1.88 (1.51–2.34)	1.67 (1.25–2.23)	2.26 (1.52–3.35)
No	REF	REF	REF
Interaction Effects:			
Lost Job Entirely × SNAP	-	-	4.69 (2.69–8.17)
Paid Hours Reduced × SNAP	-	-	2.17 (1.26–3.73)
Anticipate Job Lost × SNAP	-	-	4.41 (2.08–11.13)

SNAP: Supplemental Nutrition Assistance Program. ^a^ Low food security includes survey participants with self-reported low and very low food security (*n* = 937). ^b^ Logistic regression model that includes only the variable of interest. ^c^ Logistic regression model that includes all variables in the table in addition to age, sex, race/ethnicity, partner status, children status, education level, income group, and survey wave number. ^d^ Logistic regression model that includes all variables included in adjusted model 1 and the interaction terms listed in the table. The reference group for all odds ratios is survey participants who had no change in employment status and are not a SNAP participant.

**Table 3:** Associations between Employment Status Change during Pandemic, SNAP Participation, and Very Low Food Security ^a^.

Variable	Unadjusted Models ^b^ OR (95% CI)	Adjusted Model 1 ^c^ OR (95% CI)	Adjusted Model 2 ^d^ OR (95% CI)
Job Status Change Due to COVID-19 Pandemic:			
Lost Job Entirely	4.18 (3.02–5.82)	3.61 (2.43–5.36)	4.58 (2.74–7.67)
Paid Hours Reduced	2.36 (1.70–3.27)	2.19 (1.48–3.26)	3.22 (2.01–5.17)
Anticipate Job Loss	1.34 (0.88–2.03)	1.71 (1.03–2.82)	2.25 (1.24–4.06)
No Change	REF	REF	REF
Current SNAP Participant:			
Yes	1.91 (1.47–2.49)	1.44 (1.03–2.03)	2.55 (1.51–4.30)
No	REF	REF	REF
Interaction Effects:			
Lost Job Entirely × SNAP	-	-	6.07 (3.38–10.90)
Paid Hours Reduced × SNAP	-	-	2.29 (1.13–4.66)
Anticipate Job Lost × SNAP	-	-	2.37 (0.90–6.24)

SNAP: Supplemental Nutrition Assistance Program. ^a^ Very low food security includes survey participants with self-reported very low food security (*n* = 300). ^b^ Logistic regression model that includes only the variable of interest. ^c^ Logistic regression model that includes all variables in the table in addition to age, sex, race/ethnicity, partner status, children status, education level, income group, and survey wave number. ^d^ Logistic regression model that includes all variables included in adjusted model 1 and the interaction terms listed in the table. The reference group for all odds ratios is survey participants who had no change in employment status and are not a SNAP participant.

**Table 4:** Stratified Regression Models Examining Associations between Employment Status Change during Pandemic and Food Security Status.

Variable	Low Food Security ^a^		Very Low Food Security ^b^	
	SNAP Participants OR (95% CI) ^c^	Non-Participants OR (95% CI)	SNAP Participants OR (95% CI)	Non-Participants OR (95% CI)
Job Status Change Due to COVID-19 Pandemic:				
Lost Job Entirely	2.15 (1.16–3.99)	2.85 (1.86–4.37)	2.82 (1.46–5.45)	4.46 (2.64–7.52)
Paid Hours Reduced	1.23 (0.66–2.32)	2.51 (1.76–3.57)	1.18 (0.53–2.63)	3.03 (1.87–4.91)
Anticipate Job Loss	2.58 (1.05–6.35)	2.63 (1.70–4.05)	1.23 (0.43–3.47)	2.11 (1.16–3.83)
No Change	REF	REF	REF	REF

SNAP: Supplemental Nutrition Assistance Program. ^a^ Outcome measure is low to very low food security status (*n* = 973). ^b^ Outcome measure is very low food security status (*n* = 300). ^c^ All logistic regression models adjusted for age, sex, race/ethnicity, partner status, children status, education level, income group, and survey wave number.

## Data Availability

Data used for this analysis can be accessed by contacting the Center for Social and Behavioral Sciences at the University of Illinois at Urbana-Champaign.
